# Effect of patient clothing removal with scissors on time to defibrillation by lay rescuers: a randomized controlled simulation trial

**DOI:** 10.1016/j.resplu.2026.101281

**Published:** 2026-02-26

**Authors:** Kentaro Omatsu, Reishin Matsuyama, Neneka Kamitani, Genjiro Muro, Azusa Tsunemoto, Gen Toyama, Eiji Hori, Yutaka Takei

**Affiliations:** a Department of Emergency Medical Sciences, Faculty of Medical Technology, Niigata University of Health and Welfare, Niigata, Japan; b Graduate School of Health and Welfare, Niigata University of Health and Welfare, Niigata, Japan

**Keywords:** Out-of-hospital cardiac arrest, Automated external defibrillator, Bystander response, AED pad placement, Simulation study

## Abstract

**Background:**

Early defibrillation using an automated external defibrillator (AED) is a key determinant of survival after out-of-hospital cardiac arrest (OHCA). However, the impact of clothing removal strategies on time to defibrillation and AED pad placement accuracy remains unclear.

**Methods:**

We conducted a prospective, randomized controlled simulation trial involving 40 undergraduate students without healthcare provider–level resuscitation training. Participants were randomly assigned (1:1) to the scissors or no-scissors group during a simulated OHCA scenario using a clothed manikin. The primary outcome was time from AED power-on to shock delivery. The secondary outcome was AED pad placement accuracy evaluated using standardized anatomical criteria.

**Results:**

The median time from AED power-on to shock delivery was longer in the scissors group than in the no-scissors group (118 vs 91.5 s; Hodges–Lehmann median difference 24 s, 95% CI 6–39; *p* = 0.004). The AED pad placement accuracy did not differ between groups (anterior pad: OR 1.00, 95% CI 0.13–7.89; lateral pad: OR 0.67, 95% CI 0.19–2.33). Overall, the correct pad placement rates were low in both groups (10% anterior, 55% lateral).

**Conclusions:**

In this randomized controlled simulation trial, the use of scissors for clothing removal was associated with a longer time from AED power-on to shock delivery, without improvement in pad placement accuracy. These findings do not support routine scissor use under the simulated conditions. Larger studies are needed to determine the role of clothing removal strategies in AED training for lay rescuers.

## Introduction

Survival after out-of-hospital cardiac arrest (OHCA) largely depends on early recognition, high-quality cardiopulmonary resuscitation (CPR),^1^ and rapid defibrillation.^2^, ^3^ Although automated external defibrillators (AEDs) have been widely disseminated, bystander defibrillation remains limited in many settings.^3^, ^4^

Simulation studies have shown that even when AEDs are accessible, lay rescuers often struggle with the sequence of steps required for AED use, resulting in delays in shock delivery.^5^ Accurate AED pad placement is frequently not achieved even under controlled simulation conditions, including among trained healthcare providers.^6^, ^7^, ^8^

Current resuscitation guidelines emphasize accurate AED pad positioning as essential for effective defibrillation. To achieve correct AED pad placement based on anatomical landmarks, both the European Resuscitation Council (ERC) and American Heart Association (AHA) guidelines recommend applying AED pads directly to bare skin and moving or removing clothing when it interferes with proper placement.^9^, ^10^ The AHA implementation guidance also anticipates the need for chest preparation during AED use and includes scissors among standard accessories for cutting clothing when necessary.^11^

Clothing management itself may influence resuscitation performance. Simulation studies report that instructions to undress the patient can delay CPR initiation, while chest exposure improves landmark recognition and clothing can affect compression quality.^12^, ^13^, ^14^ These findings suggest that chest exposure may influence rescuer performance, but the effect of specific clothing removal strategies during AED use remains unclear.

Therefore, this study aimed to examine whether the use of scissors to cut clothing affects (1) time from AED power-on to shock delivery and (2) AED pad placement accuracy in a simulated OHCA scenario.

## Methods

### Study design

This prospective, parallel-group, randomized controlled simulation trial was conducted at Niigata University of Health and Welfare (Niigata, Japan). The study was approved by the Ethics Committee of Niigata University of Health and Welfare (Approval No. 19577-250702), and written informed consent was obtained from all participants prior to enrollment. The trial was registered in the University Hospital Medical Information Network (UMIN) Clinical Trials Registry (UMIN-CTR; UMIN000057995; https://center6.umin.ac.jp/cgi-open-bin/ctr/ctr_view.cgi?recptno=R000066298). This study was reported in accordance with the CONSORT 2025 statement.^15^

### Participants

Participants were undergraduate students aged 18–24 years enrolled at Niigata University of Health and Welfare (Niigata, Japan). Participants were recruited from health- and welfare-related academic programs, including Nursing, Prosthetics and Orthotics, Physical Therapy, Health and Sports Sciences, Psychology, Social Welfare, and Acupuncture.

In Japan, undergraduate nursing students are not required to complete healthcare provider–level basic life support (BLS) certification prior to clinical practice. Some participants may have received brief lay-rescuer–level instruction (e.g., driver’s license courses or introductory classes), but none had completed provider-level certification (e.g., AHA BLS provider course) at the time of participation. Prior experience using scissors was not assessed in this study.

At enrollment, participants completed a written questionnaire documenting their age, sex, and prior resuscitation training history, and provided written informed consent. The form also advised participants to refrain from participation if they had any injuries or medical conditions that could interfere with the simulation. No participants declined participation for these reasons.

### Randomization

After completion of enrollment and informed consent, participants were allocated in a 1:1 ratio using a concealed lottery-based method.^16^ Forty identical sticks were prepared in advance, with 20 marked for each group, and placed in an opaque container. The container was shaken before each draw, and each participant drew one stick immediately before the start of the simulation scenario to determine group allocation. The markings were not visible prior to drawing. The procedure was supervised by the first author (K.O.), who was not involved in outcome assessment. Owing to the nature of the intervention, participants and investigators were not blinded to group allocation.

### Intervention and procedure

Each participant performed a simulated AED use scenario on an adult manikin (Resusci Anne Simulator, Laerdal, Norway). The manikin wore two clothing layers: a short-sleeved polyester T-shirt and a long-sleeved cotton–polyester buttoned shirt with all buttons fastened. Clothing cut during the scenario was replaced with new clothing for each subsequent participant. An AED trainer (Laerdal AED Trainer 2, Laerdal, Norway) was used to simulate AED operation. The AED trainer was programmed with Japanese voice prompts, consistent with the AEDs commonly used in public settings in Japan. A shockable rhythm was preselected, and participants were required to follow the AED prompts through pad application and shock delivery. The AED trainer was placed 3 m from the manikin to simulate retrieval in a single-rescuer scenario. Both the AED trainer and manikin were placed in the same simulation room. No AED signage or directional cues were provided, and participants were able to visually identify the AED trainer at the start of the scenario. The AED pads were not replaced with new pads for each participant. However, the adhesive condition of the pads was checked before each simulation, and AED pads were immediately replaced if reduced adhesion was observed. No instances of AED pad detachment or peeling occurred during simulation in any participant. Participants in the scissors group were provided standard stainless steel trauma scissors (SÖHNGEN® Rescue Scissors, Germany). Participants in the no-scissors group were instructed not to use scissors but were allowed to expose the chest using any other method (e.g., unbuttoning, lifting, or pulling clothing) at their discretion.

All participants were instructed to deliver the first shock as quickly as possible by following AED voice prompts (Supplementary Appendix). Immediately before the simulation, participants received group-specific instructions. Participants in the no-scissors group were instructed to deliver an electrical shock as quickly as possible using the AED and were not provided with scissors. Participants in the scissors group were instructed to cut the clothing using scissors and to use the AED as quickly as possible. All participants in the scissors group initially used the scissors; however, some subsequently used alternative methods such as tearing clothing during the scenario. No additional hands-on instruction or demonstration regarding clothing removal techniques was provided by the investigators beyond the AED voice prompts.

### Outcomes

The primary outcome was the time from AED power-on to shock delivery, measured in seconds. Time intervals were measured in real time using a digital stopwatch by a single investigator who was present in the simulation room and was not involved in randomization or participant instruction. The secondary outcome was AED pad placement accuracy, which was evaluated using standardized anatomical criteria based on a previously validated simulation study.^7^, ^8^, ^9^ Still photographs were taken immediately after pad application, and pad placement accuracy was independently assessed by two investigators. AED pad placement was assessed separately for the anterior and lateral pads according to the predefined anatomical landmarks (Table 1). In cases of disagreement, a third investigator was designated to adjudicate.

**Table 1:** Criteria for AED pad placement evaluation.

	**Anterior pad criteria**	**Lateral pad criteria**
1	The inner edge should not cross the midline	The inner edge should not cross the midline
2	The upper edge should not exceed the crest of the trapezius	The upper edge should not exceed the nipple line
3	The lower edge should not extend below the nipple line	The lower edge should not extend below the umbilical line

### Statistical analysis

Continuous variables are presented as median (interquartile range [IQR]) and were compared using the Wilcoxon rank-sum test due to non-normal distribution. The Hodges–Lehmann estimator was used to estimate the median difference with 95% confidence intervals (CI). Categorical variables were analyzed using Fisher’s exact test. For secondary outcomes, odds ratios (ORs) with 95% CIs were calculated to quantify between-group differences.

No formal a priori sample size calculation was performed, as the study was designed as an exploratory simulation trial to estimate effect sizes for future adequately powered studies.

A two-sided *p* < 0.05 was considered statistically significant. Analyses were performed using JMP Pro 18 (SAS Institute, Cary, NC, USA).

## Results

### Participant characteristics

A total of 40 undergraduate students from non-emergency medical technician programs participated: 16 from the Department of Health and Sports Sciences, seven from Nursing, seven from Physical Therapy, three from Psychology, five from Prosthetics and Orthotics, one from Social Welfare, and one from Acupuncture.

Although most participants had previously attended basic CPR/AED courses, their prior training did not include specific instruction on clothing removal techniques or the use of trauma scissors. None of the participants had completed healthcare provider–level resuscitation training. All participants completed the trial and were included in the analysis. Baseline characteristics are summarized in Table 2. No adverse events occurred during the simulation.

**Table 2:** Baseline characteristics of participants.

**Characteristics**	**No-scissors group (*n* = 20)**	**Scissors group (*n* = 20)**
Age, years, median (IQR)	20 (20–21)	20 (19–21)
Male sex, *n* (%)	10 (50%)	8 (40%)
Prior Lay CPR/AED training, *n* (%)	18 (90%)	16 (80%)
Training ≥6 months before, *n* (%)*	16 (80%)	17 (85%)
Training <6 months before, *n* (%)*	4 (20%)	3 (15%)
**Academic program**		
Nursing	4 (20.0%)	3 (15.0%)
Prosthetics and Orthotics	3 (15.0%)	2 (10.0%)
Health and Sports Sciences	8 (40.0%)	8 (40.0%)
Social Welfare	1 (5.0%)	0 (0.0%)
Psychology	0 (0.0%)	3 (15.0%)
Physical Therapy	3 (15.0%)	4 (20.0%)
Acupuncture	1 (5.0%)	0 (0.0%)

*Abbreviations:* IQR, interquartile range; CPR, cardiopulmonary resuscitation; AED, automated external defibrillator.

Data are presented as median (IQR) or number (%), as appropriate.

* Percentages calculated among participants with prior CPR/AED training.

### Primary outcome: time from AED power-on to shock delivery (Fig. 1)

The median time from AED power-on to shock delivery was 118 s (IQR 92.3–132.8) in the scissors group and 91.5 s (IQR 50.3–101.5) in the no-scissors group. The Hodges–Lehmann estimated median difference between groups was 24 s (95% CI, 6–39 s; *p* = 0.004).

**Fig. 1:**
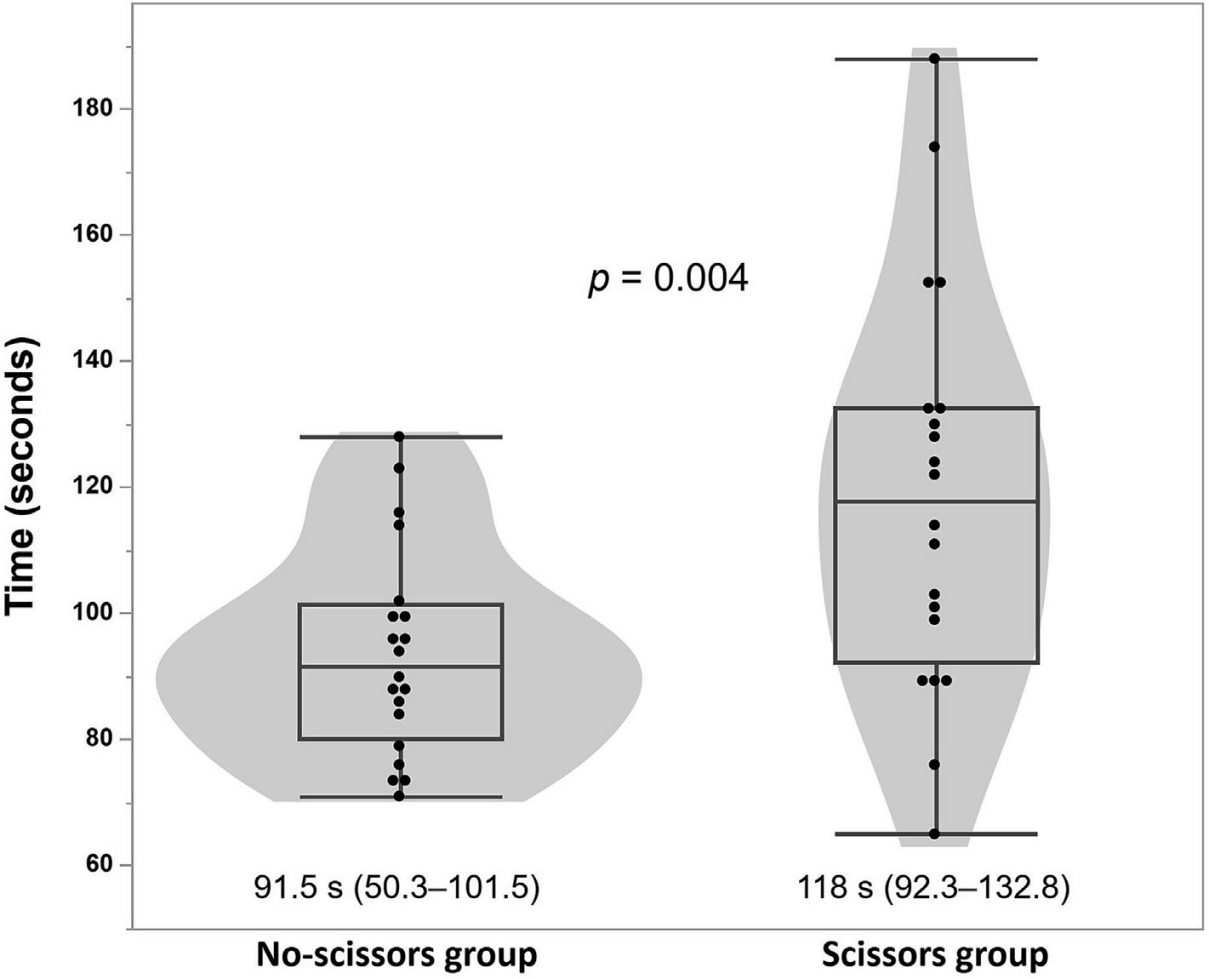
**Time from AED power-on to shock delivery**. *Abbreviation:* AED, automated external defibrillator. Violin plots with overlaid box-and-whisker plots show the distribution of time from AED power-on to shock delivery in the scissors and no-scissors groups. The dots represent individual participants. Boxes indicate the median and interquartile range, and whiskers indicate the minimum and maximum values. Values below each group are presented as median (interquartile range). The *p*-values were calculated using the Wilcoxon rank-sum test.

### Secondary outcome: AED Pad placement accuracy (Table 3)

No significant differences were observed in the AED pad placement accuracy between the groups. For anterior pad placement, correct positioning occurred in 2 of 20 participants (10%) in both the scissors and no-scissors groups (OR 1.00, 95% CI 0.13–7.89; *p* = 1.0). For lateral pad placement, correct positioning occurred in 10 of 20 participants (50%) in the scissors group and 12 of 20 participants (60%) in the no-scissors group (OR 0.67, 95% CI 0.19–2.33; *p* = 0.75). Overall, correct AED pad placement rates were low across participants (10% for anterior pads and 55% for lateral pads). No disagreements occurred between the two assessors in the evaluation of AED pad placement accuracy.

**Table 3:** AED pad placement accuracy according to the use of scissors.

**Pad location**	**No-scissors group (*n* = 20)**	**Scissors group (*n* = 20)**	**Odds ratio (95% CI)**	***p*-value**
Anterior pad				1.0
Correct	2 (10%)	2 (10%)	1.00 (0.13–7.89)	
Incorrect	18 (90%)	18 (90%)		
Lateral pad				0.75
Correct	12 (60%)	10 (50%)	0.67 (0.19–2.33)	
Incorrect	8 (40%)	10 (50%)		

*Abbreviations:* AED, automated external defibrillator; CI, Confidence Interval.

Odds ratios with 95% CIs were calculated. *p*-values were derived using Fisher’s exact test.

## Discussion

In this randomized controlled simulation trial, undergraduate students’ use of scissors for clothing removal was associated with a delay in shock delivery, without an improvement in AED pad placement accuracy. These findings indicate that the clothing removal strategy influenced procedural time but was not associated with measurable differences in pad positioning in this simulated scenario.

Previous simulation studies have reported delayed initiation of CPR when clothing removal is required before resuscitation actions.^12^ In the present study, assignment to cut clothing was likewise associated with a longer time to shock delivery. The distribution of time to shock delivery also differed between groups, with greater variability observed in the no-scissors group (Fig. 1). Trauma simulation studies have also shown that patient exposure time differs depending on removal methods and tools used.^17^, ^18^ The longer time observed in the scissors group is consistent with these observations, although the present study did not evaluate exposure quality.

The AED pad placement accuracy was low in both groups, consistent with prior simulation studies showing substantial variability in AED pad placement among lay rescuers despite standardized instructions.^6^, ^7^, ^8^ In contrast to several prior studies^6^, ^7^, ^8^ that reported lower accuracy for the lateral pad, inaccurate placement in our study was also frequently observed for the anterior pad. This difference may be related to the use of unclothed manikins in many previous simulations.

In this simulation involving undergraduate students representing lay rescuers, routine use of scissors before pad application did not improve pad placement accuracy and was associated with a longer time to shock delivery. These findings do not support routine scissor use under the tested conditions, although cutting tools are commonly included in AED kits.^11^ These findings support the need for larger studies to clarify the optimal clothing management during AED use and AED training for lay rescuers.

### Study limitations

This study has several limitations. First, it was a simulation-based study using a manikin, which cannot fully replicate the psychological stress, environmental constraints, or social dynamics encountered during actual cardiac arrest events. Second, the sample size was determined pragmatically based on feasibility considerations, and no formal a priori power calculation was performed. Although a statistically significant difference was observed for the primary outcome, it may have been underpowered to detect smaller differences in AED pad placement accuracy, and the null findings for the secondary outcome should therefore be interpreted cautiously. Third, the primary outcome was measured in real time by a single investigator without video recording. Although time measurement was based on clearly defined procedural events, this approach may introduce measurement bias. Fourth, the participants were undergraduate students enrolled in health- or welfare-related academic programs and may not be representative of the general lay population. Accordingly, the generalizability of the findings to other bystander groups or real-world settings is limited. Finally, prior experience with trauma scissors was not formally assessed. Variability in informal exposure to scissors in other contexts (e.g., routine academic or clinical activities) may therefore have existed and could have influenced performance.

## Conclusion

In this randomized controlled simulation trial, the use of scissors for clothing removal by undergraduate students was associated with a longer time from AED power-on to shock delivery, without any observed improvement in AED pad placement accuracy. Under the simulated conditions, routine scissor use may not be necessary. Larger, adequately powered studies are needed to determine whether and how specific clothing removal strategies should be incorporated into AED training for lay rescuers.

## Data sharing

The data supporting the findings of this study are available from the corresponding author upon reasonable request.

## Declaration of Generative AI and AI-assisted technologies in the writing process

During the preparation of this work, the authors used ChatGPT for English language editing and stylistic refinement. After using this tool, the authors reviewed and edited the content as needed and take full responsibility for the content of the publication.

## CRediT authorship contribution statement

**Kentaro Omatsu:** Conceptualization, Methodology, Formal analysis, Writing – original draft, Visualization, Project administration, Funding acquisition. **Reishin Matsuyama:** Investigation, Data curation, Writing – original draft. **Neneka Kamitani:** Investigation, Data curation, Writing – original draft. **Genjiro Muro:** Investigation, Data curation, Writing – original draft. **Azusa Tsunemoto:** Investigation, Data curation, Writing – original draft. **Gen Toyama:** Validation, Writing – review & editing. **Eiji Hori:** Validation, Writing – review & editing. **Yutaka Takei:** Validation, Writing – review & editing, Supervision.

## Funding

This study was supported by individual research funds from 10.13039/100015061Niigata University of Health and Welfare and by a JSPS KAKENHI grant (Grant Number JP23K02268).

## Declaration of competing interest

The authors declare no conflicts of interest.
