# Supplementary material for: Effect of patient clothing removal with scissors on time to defibrillation by lay rescuers: a randomized controlled simulation trial
Source: Resusc Plus. 2026 Feb 26;28:101281. doi: 10.1016/j.resplu.2026.101281 (PMC13000700; doi:10.1016/j.resplu.2026.101281)
Supplement: Supplementary Data 1 [file mmc1.docx]

The CONSORT reporting checklist

For checking that reports of randomised trials can be understood and used by everyone

| 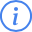 Note |
| --- |
| If you have not used a reporting guideline before, read about [how and why to use them](https:/resources.equator-network.org/about/reporting-guidelines.html) and check whether CONSORT is the [most applicable reporting guideline](https:/resources.equator-network.org/reporting-guidelines/consort/index.html?#applicability) for your work.  Reporting guidelines are most useful when used early in research. When writing a manuscript or application, consider using the [Full Guidance](https:/resources.equator-network.org/reporting-guidelines/consort/index.html) where you’ll see explanations and examples for each item.  After writing, demonstrate adherence by completing this checklist:   1. Specify where each item is described (see [Note 1](#sec-specify)). 2. Cite this checklist (See [Note 2](#sec-cite)). 3. Include your completed checklist as a supplement when submitting to a journal so that future readers can use it to find information. |

|  | Item Description | Location (or reason for not reporting) |
| --- | --- | --- |
| **Title and Abstract** |  |  |
| [1a. Title](https:/resources.equator-network.org/reporting-guidelines/consort/items/title.html?utm_source=consort&utm_medium=checklist&utm_campaign=CONSORT_2025_v1_1) | Identification as a randomised trial. | Page 1, Line1–2 |
| [1b. Structured Abstract](https:/resources.equator-network.org/reporting-guidelines/consort/items/structured-abstract.html?utm_source=consort&utm_medium=checklist&utm_campaign=CONSORT_2025_v1_1) | Structured summary of the trial design, methods, results, and conclusions. | Page1–2 |
| **Open Science** |  |  |
| [2. Trial Registration](https:/resources.equator-network.org/reporting-guidelines/consort/items/trial-registration.html?utm_source=consort&utm_medium=checklist&utm_campaign=CONSORT_2025_v1_1) | Name of trial registry, identifying number (with URL) and date of registration. | Page 4, Line 70–71 |
| [Protocol and statistical analysis plan](https:/resources.equator-network.org/reporting-guidelines/consort/items/protocol-and-statistical-analysis-plan.html?utm_source=consort&utm_medium=checklist&utm_campaign=CONSORT_2025_v1_1) | Where the trial protocol and statistical analysis plan can be accessed. | The study protocol and statistical analysis plan are available from the corresponding author upon reasonable request. |
| [4. Data sharing](https:/resources.equator-network.org/reporting-guidelines/consort/items/data-sharing.html?utm_source=consort&utm_medium=checklist&utm_campaign=CONSORT_2025_v1_1) | Where and how the individual de-identified participant data (including data dictionary), statistical code and any other materials can be accessed. | Page 14–15, Line 250–252 |
| 5. Funding and Conflicts of Interest |  |  |
| [5a. Funding](https:/resources.equator-network.org/reporting-guidelines/consort/items/funding.html?utm_source=consort&utm_medium=checklist&utm_campaign=CONSORT_2025_v1_1) | Sources of funding and other support (eg, supply of drugs), and role of funders in the design, conduct, analysis, and reporting of the trial. | Page 14, Line 243–245 |
| [5b. Conflicts of interest](https:/resources.equator-network.org/reporting-guidelines/consort/items/conflicts-of-interest.html?utm_source=consort&utm_medium=checklist&utm_campaign=CONSORT_2025_v1_1) | Financial and other conflicts of interest of the manuscript authors. | Page 14, Line 247–248 |
| **Introduction** |  |  |
| [6. Background and rationale](https:/resources.equator-network.org/reporting-guidelines/consort/items/background-and-rationale.html?utm_source=consort&utm_medium=checklist&utm_campaign=CONSORT_2025_v1_1) | Scientific background and rationale. | Page 3–4, Line 37–58 |
| [7. Objectives](https:/resources.equator-network.org/reporting-guidelines/consort/items/objectives.html?utm_source=consort&utm_medium=checklist&utm_campaign=CONSORT_2025_v1_1) | Specific objectives related to benefits and harms. |  |
| **Methods** |  | Page 4, Line 59–61 |
| [8. Patient and public involvement](https:/resources.equator-network.org/reporting-guidelines/consort/items/patient-and-public-involvement.html?utm_source=consort&utm_medium=checklist&utm_campaign=CONSORT_2025_v1_1) | Details of patient or public involvement in the design, conduct and reporting of the trial. | Patients or members of the public were not involved in the design, conduct, reporting, or dissemination plans of this simulation study. |
| [9. Trial Design](https:/resources.equator-network.org/reporting-guidelines/consort/items/trial-design.html?utm_source=consort&utm_medium=checklist&utm_campaign=CONSORT_2025_v1_1) | Description of trial design including type of trial (eg, parallel group, crossover), allocation ratio, and framework (eg, superiority, equivalence, non-inferiority, exploratory). | Page 4, Line 65 |
| [10. Changes to trial protocol](https:/resources.equator-network.org/reporting-guidelines/consort/items/changes-to-trial-protocol.html?utm_source=consort&utm_medium=checklist&utm_campaign=CONSORT_2025_v1_1) | Important changes to the trial after it commenced including any outcomes or analyses that were not pre-specified, with reason. | There were no important changes to the trial design, outcomes, or analyses after trial commencement. All outcomes and analyses were pre-specified in the registered protocol. |
| [11. Trial Setting](https:/resources.equator-network.org/reporting-guidelines/consort/items/trial-setting.html?utm_source=consort&utm_medium=checklist&utm_campaign=CONSORT_2025_v1_1) | Settings (eg, community, hospital) and locations (eg, countries, sites) where the trial was conducted. | Page 4, Line 66 |
| 12. Eligibility Criteria |  |  |
| [12a. Participants](https:/resources.equator-network.org/reporting-guidelines/consort/items/eligibility-criteria-participants.html?utm_source=consort&utm_medium=checklist&utm_campaign=CONSORT_2025_v1_1) | Eligibility criteria for participants. | Page 5, Line 74–84 |
| [12b. Other](https:/resources.equator-network.org/reporting-guidelines/consort/items/eligibility-criteria-sites-individuals.html?utm_source=consort&utm_medium=checklist&utm_campaign=CONSORT_2025_v1_1) | If applicable, eligibility criteria for sites and for individuals delivering the interventions (eg, surgeons, physiotherapists). | The intervention was performed solely by participants (lay rescuers), and no specific eligibility criteria for intervention providers were applicable. |
| [13. Intervention and comparator](https:/resources.equator-network.org/reporting-guidelines/consort/items/intervention-and-comparator.html?utm_source=consort&utm_medium=checklist&utm_campaign=CONSORT_2025_v1_1) | Intervention and comparator with sufficient details to allow replication. If relevant, where additional materials describing the intervention and comparator (eg, intervention manual) can be accessed. | Page 6–8, Line 101–132 |
| [14. Outcomes](https:/resources.equator-network.org/reporting-guidelines/consort/items/outcomes.html?utm_source=consort&utm_medium=checklist&utm_campaign=CONSORT_2025_v1_1) | Prespecified primary and secondary outcomes, including the specific measurement variable (eg, systolic blood pressure), analysis metric (eg, change from baseline, final value, time to event), method of aggregation (eg, median, proportion), and time point for each outcome. | Page 8–9, Line 134–144 |
| [15. Harms](https:/resources.equator-network.org/reporting-guidelines/consort/items/harms.html?utm_source=consort&utm_medium=checklist&utm_campaign=CONSORT_2025_v1_1) | How harms were defined and assessed (eg, systematically, non-systematically). | No harms or adverse events were anticipated or assessed, as this study involved a non-invasive manikin-based simulation without clinical intervention. |
| 16. Sample Size |  |  |
| [16a. How sample size was determined](https:/resources.equator-network.org/reporting-guidelines/consort/items/sample-size-determination.html?utm_source=consort&utm_medium=checklist&utm_campaign=CONSORT_2025_v1_1) | How sample size was determined, including all assumptions supporting the sample size calculation. | Page 9, Line 153–155 |
| [16b. Interim analyses and stopping criteria](https:/resources.equator-network.org/reporting-guidelines/consort/items/sample-size-interim-analyses-and-stopping-guidelines.html?utm_source=consort&utm_medium=checklist&utm_campaign=CONSORT_2025_v1_1) | Explanation of any interim analyses and stopping guidelines. | No interim analyses or stopping rules were specified or performed, as the study had a fixed sample size. |
| 17. Randomisation |  |  |
| [17a. Sequence Generation](https:/resources.equator-network.org/reporting-guidelines/consort/items/randomisation-sequence-generation.html?utm_source=consort&utm_medium=checklist&utm_campaign=CONSORT_2025_v1_1) | Who generated the random allocation sequence and the method used. | Page 9, Line 153–155 |
| [17b. Type of Randomisation](https:/resources.equator-network.org/reporting-guidelines/consort/items/randomisation-type-of-randomisation.html?utm_source=consort&utm_medium=checklist&utm_campaign=CONSORT_2025_v1_1) | Type of randomisation and details of any restriction (eg, stratification, blocking, and block size). | Page 9, Line 92–97 |
| [18. Allocation concealment mechanism](https:/resources.equator-network.org/reporting-guidelines/consort/items/allocation-concealment-mechanism.html?utm_source=consort&utm_medium=checklist&utm_campaign=CONSORT_2025_v1_1) | Mechanism used to implement the random allocation sequence (eg, central computer/telephone; sequentially numbered, opaque, sealed containers), describing any steps to conceal the sequence until interventions were assigned. | Page 9, Line 93–96 |
| [19. Implementation](https:/resources.equator-network.org/reporting-guidelines/consort/items/implementation.html?utm_source=consort&utm_medium=checklist&utm_campaign=CONSORT_2025_v1_1) | Whether the personnel who enrolled and those who assigned participants to the interventions had access to the random allocation sequence. | Page 9, Line 95–99 |
| 20. Blinding |  |  |
| [20a. Who was blinded](https:/resources.equator-network.org/reporting-guidelines/consort/items/blinding-who.html?utm_source=consort&utm_medium=checklist&utm_campaign=CONSORT_2025_v1_1) | Who was blinded after assignment to interventions (eg, participants, care providers, outcome assessors, data analysts). | Page 9, Line 98–99 |
| [20b. How blinding was achieved](https:/resources.equator-network.org/reporting-guidelines/consort/items/blinding-how.html?utm_source=consort&utm_medium=checklist&utm_campaign=CONSORT_2025_v1_1) | If blinded, how blinding was achieved and description of the similarity of interventions. | Page 9, Line 98–99 |
| 21. Statistical methods |  |  |
| [21a. Comparing groups](https:/resources.equator-network.org/reporting-guidelines/consort/items/statistical-methods-comparing-groups-primary-secondary-outcomes-harms.html?utm_source=consort&utm_medium=checklist&utm_campaign=CONSORT_2025_v1_1) | Statistical methods used to compare groups for primary and secondary outcomes, including harms. | Page 9, Line 47–52 |
| [21b. Definition of who is included in each analysis](https:/resources.equator-network.org/reporting-guidelines/consort/items/statistical-methods-definition-of-who-is-included-in-each-analysis.html?utm_source=consort&utm_medium=checklist&utm_campaign=CONSORT_2025_v1_1) | Definition of who is included in each analysis (e.g., all randomised participants), and in which group. | All randomized participants were included in the analysis according to their assigned group |
| [21c. Missing Data](https:/resources.equator-network.org/reporting-guidelines/consort/items/statistical-methods-missing-data.html?utm_source=consort&utm_medium=checklist&utm_campaign=CONSORT_2025_v1_1) | How missing data were handled in the analysis. | There were no missing data; therefore, no imputation methods were applied. |
| [21d. Additional Analyses](https:/resources.equator-network.org/reporting-guidelines/consort/items/statistical-methods-additional-analyses.html?utm_source=consort&utm_medium=checklist&utm_campaign=CONSORT_2025_v1_1) | Methods for any additional analyses (eg, subgroup and sensitivity analyses), distinguishing pre-specified from post hoc. | No subgroup, sensitivity, or post hoc analyses were performed. |
| 22. Participant flow, including flow diagram |  |  |
| [22a. Participant Numbers](https:/resources.equator-network.org/reporting-guidelines/consort/items/participant-flow-numbers.html?utm_source=consort&utm_medium=checklist&utm_campaign=CONSORT_2025_v1_1) | For each group, the numbers of participants who were randomly assigned, received intended intervention, and were analysed for the primary outcome. | Page 9–10, Line 160–164 |
| [22b. Losses and exclusions](https:/resources.equator-network.org/reporting-guidelines/consort/items/participant-flow-losses-and-exclusions.html?utm_source=consort&utm_medium=checklist&utm_campaign=CONSORT_2025_v1_1) | For each group, losses and exclusions after randomisation, together with reasons. | There were no losses or exclusions after randomization. |
| 23. Recruitment |  |  |
| [23a. Dates](https:/resources.equator-network.org/reporting-guidelines/consort/items/recruitment-dates.html?utm_source=consort&utm_medium=checklist&utm_campaign=CONSORT_2025_v1_1) | Dates defining the periods of recruitment and follow-up for outcomes of benefits and harms. | Participants were recruited between July 2025 and September 2025. Outcome assessment was completed immediately after the simulation scenario. |
| [23b. Reasons for stopping recruitment](https:/resources.equator-network.org/reporting-guidelines/consort/items/recruitment-why-stopped.html?utm_source=consort&utm_medium=checklist&utm_campaign=CONSORT_2025_v1_1) | If relevant, why the trial ended or was stopped. | Recruitment ended after the predefined sample size was reached. |
| 24. Intervention and comparator delivery |  |  |
| [24a. As Administered](https:/resources.equator-network.org/reporting-guidelines/consort/items/intervention-comparator-delivery-as-administered.html?utm_source=consort&utm_medium=checklist&utm_campaign=CONSORT_2025_v1_1) | Intervention and comparator as they were actually administered (eg, where appropriate, who delivered the intervention/comparator, whether participants adhered, whether they were delivered as intended (fidelity)). | The interventions were delivered as intended. All participants followed the assigned instructions, and all completed the simulation scenario according to protocol. |
| [24b. Concomitant Care](https:/resources.equator-network.org/reporting-guidelines/consort/items/intervention-comparator-delivery-concomitant-care.html?utm_source=consort&utm_medium=checklist&utm_campaign=CONSORT_2025_v1_1) | Concomitant care received during the trial for each group. | No additional care or co-interventions were provided during the simulation. |
| [25. Baseline Data](https:/resources.equator-network.org/reporting-guidelines/consort/items/baseline-data.html?utm_source=consort&utm_medium=checklist&utm_campaign=CONSORT_2025_v1_1) | A table showing baseline demographic and clinical characteristics for each group. | Table 2 |
| [26. Numbers analysed, outcomes, and estimation](https:/resources.equator-network.org/reporting-guidelines/consort/items/numbers-analysed-outcomes-estimation.html?utm_source=consort&utm_medium=checklist&utm_campaign=CONSORT_2025_v1_1) | For each primary and secondary outcome, by group:   - the number of participants included in the analysis. - the number of participants with available data at the outcome time point. - result for each group, and the estimated effect size and its precision (such as 95% confidence interval). - for binary outcomes, presentation of both absolute and relative effect size. | Page 9–11, Line 161–187 |
| [27. Harms](https:/resources.equator-network.org/reporting-guidelines/consort/items/harms.html?utm_source=consort&utm_medium=checklist&utm_campaign=CONSORT_2025_v1_1) | All harms or unintended events in each group. | Page 10, Line 169–170 |
| [28. Ancillary Analyses](https:/resources.equator-network.org/reporting-guidelines/consort/items/ancillary-analyses.html?utm_source=consort&utm_medium=checklist&utm_campaign=CONSORT_2025_v1_1) | Any other analyses performed, including subgroup and sensitivity analyses, distinguishing pre-specified from post hoc. | No subgroup, sensitivity, or other ancillary analyses were performed. |
| **Discussion** |  |  |
| [29. Interpretation](https:/resources.equator-network.org/reporting-guidelines/consort/items/interpretation.html?utm_source=consort&utm_medium=checklist&utm_campaign=CONSORT_2025_v1_1) | Interpretation consistent with results, balancing benefits and harms, and considering other relevant evidence. | Page 12, Line 190–214 |
| [30. Limitations](https:/resources.equator-network.org/reporting-guidelines/consort/items/limitations.html?utm_source=consort&utm_medium=checklist&utm_campaign=CONSORT_2025_v1_1) | Trial limitations, addressing sources of potential bias, imprecision, generalisability, and, if relevant, multiplicity of analyses. | Page 13, Line 216–233 |

## 1 How to specify where content is

Tell the reader where they can find information. E.g.,

- Results; paragraph 2
- Methods, Participants; paragraphs 1 & 2.
- Table 3
- Supplement B, para. 4

If you have chosen not to describe an item, explain why. You can do this in the checklist, or as a note below it.

You can describe items in the article body, or in tables, figures, or supplementary materials, and should prioritize items you feel are most important to your intended audience. The order of items in your manuscript does not need to match the order of items in this checklist. You can decide how best to structure your work.

## 2 How to cite

Describe how you used CONSORT at the end of your Methods section, referencing the resources you used e.g.,

‘We used the CONSORT reporting guideline(1) to draft this manuscript, and the CONSORT reporting checklist(2) when editing, included in supplement A’

If you use a reporting checklist, remember to include it as a supplement when publishing so that readers can easily find information and see how you have interpreted the guidance.

1. Hopewell S, Chan AW, Collins GS, Hróbjartsson A, Moher D, Schulz KF, et al. CONSORT 2025 statement: Updated guideline for reporting randomised trials. The BMJ [Internet]. 2025 Apr;389:e081123. Available from: <https://www.ncbi.nlm.nih.gov/pmc/articles/PMC11995449/>

2. Hopewell S, Chan AW, Collins GS, Hróbjartsson A, Moher D, Schulz KF, et al. The CONSORT reporting checklist. In: Harwood J, Albury C, Beyer J de, Schlüssel M, Collins G, editors. The EQUATOR network reporting guideline platform [Internet]. The UK EQUATOR Centre; 2025. Available from: [https:/resources.equator-network.org/reporting-guidelines/consort/consort-checklist.docx](https://https:/resources.equator-network.org/reporting-guidelines/consort/consort-checklist.docx)
