# Supplementary material for: Effect of patient clothing removal with scissors on time to defibrillation by lay rescuers: a randomized controlled simulation trial
Source: Resusc Plus. 2026 Feb 26;28:101281. doi: 10.1016/j.resplu.2026.101281 (PMC13000700; doi:10.1016/j.resplu.2026.101281)
Supplement: Supplementary Data 2 [file mmc2.docx]

**Supplementary Appendix 1. AED voice prompts used in the simulation**

The following voice prompts were delivered by the AED trainer (Laerdal AED Trainer 2, Laerdal, Norway) in Japanese during the simulation scenario. Descriptions of participants’ actions are provided in brackets for clarity and were not delivered as voice prompts. These prompts reflect the default configuration of the AED trainer and may differ from those used in other AED models.

1. （起動音）「ピー」

(“Beep sound.”)

[Participant pressed the power button.]

2. 「パッドを胸に装着してください。」

(“Apply the pads to the chest.”)

3. 「ランプが光っているソケットにパッドのコネクターを接続してください。」

(“Connect the pad connector to the flashing socket.”)

[Participant connected the pad connector.]

4. 「心電図を解析中です。からだに触れないでください。」

(“Analyzing heart rhythm. Do not touch the patient.”)

5. 「ショックが必要です。充電中です。からだから離れてください。」

(“Shock advised. Charging. Stand clear of the patient.”)

6. 「ピー」

(“Beep sound.”)

7. 「ショックを実行します。オレンジボタンを押してください。」

(“Shock will be delivered. Press the orange button.”)

[Participant pressed the shock button.]

8. 「ショックが完了しました。一時中断中です。ただちに胸骨圧迫と人工呼吸をしてください。」

(“Shock delivered. Paused. Start chest compressions and rescue breaths immediately.”)
